# Characteristics of Dermatology Residency Program Morbidity and Mortality Conferences: A Survey of Program Directors

**DOI:** 10.2196/45194

**Published:** 2023-07-31

**Authors:** Carolina V Alexander-Savino, Dean S Morrell, Julie E Mervak, Edith V Bowers

**Affiliations:** 1 State University of New York Upstate Medical University Syracuse, NY United States; 2 Department of Dermatology University of North Carolina at Chapel Hill Chapel Hill, NC United States; 3 Department of Dermatology University of Michigan Ann Arbor, MI United States

**Keywords:** morbidity and mortality conference, MMC, dermatology residency, Accreditation Council of Graduate Medical Education, ACGME, medical education, graduate medical education, quality improvement, health quality, quality assurance

## Introduction

Quality improvement (QI) education is important for physician training and is a common program requirement set by the Accreditation Council of Graduate Medical Education (ACGME). Some programs use morbidity and mortality conferences (MMCs) as a traditional forum to address medical errors and adverse events. However, little is known about MMCs in dermatology. Understanding how programs approach the ACGME QI requirement can provide insight into the role that QI education plays in dermatology training. Thus, this study aimed to assess how dermatology residency programs meet the ACGME QI requirement, with a focus on the characteristics of MMCs.

## Methods

An anonymous, voluntary, open survey was developed with Qualtrics [1], pilot-tested among the authors for validity, and sent to residency program directors through the Association of Professors of Dermatology’s listserve [2] from December 2021 to February 2022. Survey questions can be found in Multimedia Appendix 1.

### Ethical Considerations

The Office of Human Research Ethics at the University of North Carolina at Chapel Hill determined that this survey was not considered human subjects research (IRB# 21-2630).

## Results

Of 138 dermatology residency programs, 53 (38%) responded to the survey. All respondents completed the survey in its entirety. Most respondents (39/53, 74%, 95% CI 62%-86%) reported that residents participate in a regularly scheduled MMC. The ACGME’s QI requirement for most programs with no MMCs was met through resident QI projects (11/14, 79%, 95% CI 47%-96%). A few used other quality assurance meetings or periodic reminders and real-time feedback. MMCs were important for QI education for 12 (86%, 95% CI 69%-100%) of the 14 respondents from programs with no MMCs versus all 39 (100%, 95 % CI 99%-100%) respondents from programs with MMCs. The primary goals for MMCs were similar and included promoting a culture of safety (24/52, 46%, 95% CI 33%-59%) or improving patient care (23/52, 44%, 95% CI 32%-58%).

MMCs were held 1 to 3 (15/39, 38%, 95% CI 25%-55%) or 4 to 6 (19/39, 49%, 95% CI 35%-68%) times per year and were not open to other staff (Table 1). Programs (27/39, 69%, 95% CI 56%-86%) most commonly had resident presenters. Prominent themes discussed are unanticipated morbidity and physician-related errors (37/39, 92%, 95% CI 89%-100%) or system-related errors (37/39, 92%, 95% CI 78%-98%). Some programs (31/39, 79%, 95% CI 66%-91%) chose topics based on their teaching value. The most discussed issues included errors or delays in diagnosis (35/39, 90%, 95% CI 81%-100% and 34/39, 87%, 95% CI 74%-96%, respectively). Other error types were widely distributed (Table 1). Regardless of the meeting characteristic or format, 36 (92%, 95% CI 78%-96%) of 39 respondents reported tangible changes in their department/division because of their MMC.

**Table 1:** Morbidity and mortality conference (MMC) characteristics across dermatology residency programs in the United States.

Characteristic		Responses, n (%; n=39)	95% CI (%)
**MMCs offered per year (n)**			
	1-3	15 (38)	25-55
	4-6	19 (49)	35-68
	≥7	5 (13)	6-27
**Nursing or other ancillary staff participation**			
	Yes	9 (23)	12-40
	No	30 (77)	N/A^a^
**Presenter**			
	Resident	27 (69)	56-86
	Other	12 (31)	N/A
**Case type (multiple answers allowed)**			
	Unanticipated morbidity	37 (95)	89-100
	Physician-related error	37 (95)	89-100
	System-related error	36 (92)	78-98
	Teaching value	31 (79)	66-91
	Unanticipated mortality	26 (66)	50-80
	Patient-related error	20 (51)	32-65
**Error type (multiple answers allowed)**			
	Error in diagnosis	35 (90)	81-100
	Delay in diagnosis	34 (87)	74-96
	Lost/mishandled specimen	30 (77)	61-88
	Inadequate monitoring	29 (74)	61-88
	Failure to act on results	29 (74)	61-88
	Transfer or handoff error	28 (72)	58-86
	Patient barriers to care	28 (72)	58-86
**Tangible changes within department due to MMC?**			
	Yes	36 (92)	78-96
	No	3 (8)	N/A

^a^N/A: not applicable.

## Discussion

This is the first study to assess MMC objectives and characteristics in US dermatology training programs and to report how programs with no MMCs fulfill the ACGME’s QI requirement. Differences in the importance of MMCs in QI education and their primary objectives could explain why some programs choose alternative methods to fulfill the ACGME’s QI requirements. While nearly all respondents with an MMC believed MMCs were important for QI education, some respondents with no MMCs disagreed. Similarly, more respondents in programs with MMCs (19/38, 50%, 95% CI 34%-65%) believed that the primary goal was to promote a culture of safety, while those with no MMCs (5/14, 36%, 95% CI 16%-61%) reported that the primary goal was likely to improve patient care (7/14, 50%, 95% CI 26%-73%).

Acknowledging mistakes and learning from them can make one feel vulnerable. Improper handling of MMCs can come across as accusatory and undermine their teaching value [3]. In fact, blaming increases the likelihood of medical errors [4], and openly admitting mistakes can cause emotional distress [3,5]. Other specialties select MMC cases based on teaching value rather than adverse events [6]. Although adverse events may have teaching value, some argue MMCs may not be as effective because it is hard to talk about medical mistakes rather than focusing on discussing interesting and unusual cases [3,7]. Discussing cases based on educational interest or teaching value may be easier, but they may not align with the scope of an MMC. In dermatology, cases were primarily selected because of unanticipated morbidity and physician- or system-related errors rather than for their teaching value. Topics like unanticipated mortality or patient-related errors may be less discussed because they may be less frequent or are underreported. Intimidating physicians may discourage dermatology residents from reporting adverse events [8]. An MMC model to promote error disclosure in dermatology has been proposed [9]. Despite differences in objectives and formats, however, 92% (36/39) of respondents reported tangible changes to their departments/divisions due to MMCs, emphasizing their importance in QI education. In conclusion, this is the first study to provide insight into the role and objectives of MMCs within dermatology.

## Appendix

Multimedia Appendix 1 Survey sent to dermatology program directors asking about morbidity and mortality conferences at their program.

## Abbreviations

ACGME: Accreditation Council of Graduate Medical Education
MMC: morbidity and mortality conference
QI: quality improvement
