# Supplementary material for: Characteristics of Dermatology Residency Program Morbidity and Mortality Conferences: A Survey of Program Directors
Source: JMIR Dermatol. 2023 Jul 31;6:e45194. doi: 10.2196/45194 (PMC10425815; doi:10.2196/45194)
Supplement: Multimedia Appendix 1 [file derma_v6i1e45194_app1.docx]

## Supplemental Table 1. Survey sent to Dermatology Program Directors asking about MMCs at their program.

| Question |
| --- |
|  |
| Do Residents in your program participate in regularly scheduled Morbidity and Mortality Conference? |
| Yes |
| No |
|  |
| If respondents answered "no" to the question above: |
|  |
| How do you fulfill ACGME’s Requirements for QI education? |
| Resident QI Project |
| Other quality assurance meeting |
| Other [free text] |
|  |
| Do you think MMCs are an important component of QI education? |
| Yes |
| No |
|  |
| If respondents have residents attending a regularly scheduled MMC: |
|  |
| How many conferences does you program hold in an academic year? |
| 1-3 |
| 4-6 |
| 7-9 |
| 10 or more |
|  |
| Are nursing or ancillary staff invited to participate?  Yes |
| No |
|  |
| Who most commonly presents?  Resident |
| Program Director |
| Faculty Leader |
| Other [free text] |
|  |
| Which of the following types of cases are presented? (Select all that apply) |
| Unanticipated mortality |
| Unanticipated morbidity |
| Physician-related error |
| Patient-related error |
| System-related error |
| Teaching value |
| Other |
|  |
| What type of errors are discussed? |
| Delay in diagnosis |
| Error in diagnosis |
| Lost/mishandled specimen |
| Inadequate monitoring |
| Failure to act on results |
| Transfer or hand-off error |
| Patient barriers to care |
| Other [free text] |
|  |
| Have MMCs resulted in any tangible changes in you department/division? |
| Yes |
| No |
|  |
| Do you think MMCs are an important component of QI education? |
| Yes |
| No |
|  |
| To all respondents: |
|  |
| ^a^In your opinion, the primary goal of an MMC is: |
| To promote a culture of safety |
| To improve patient care |
| Education |
| To fulfill an academic requirement |
| Quality assurance |
| Not clear |
| Other [free text] |
|  |
|  |

MMC= Morbidity and Mortality Conference

QI= Quality Improvement

1. Question adapted from Harbison SP, Regehr G. Faculty and resident opinions regarding the role of morbidity and mortality conference. *American journal of surgery*. Feb 1999;177(2):136-9. doi:10.1016/s0002-9610(98)00319-5

Note: Respondents were able to go back and fourth between questions. No incentives were provided to respond to this survey. Monitors for multiple submissions, bot detection, security scan monitors, RelevantID and indexing prevention options were turned on in Qualtrics. Responses were anonymized.
